# Ratoon rice research: Review and prospect for the tropics

**DOI:** 10.1016/j.fcr.2024.109414

**Published:** 2024-06-15

**Authors:** Kazuki Saito, Elliott Ronald Dossou-Yovo, Ali Ibrahim

**Affiliations:** a Africa Rice Center (AfricaRice), 01 B.P. 2551, Bouake 01, Cote d’Ivoire; b International Rice Research Institute (IRRI), DAPO Box 7777, Metro Manila 1301, Philippines; c Africa Rice Center (AfricaRice), PMB 82, Abuja 901101, Nigeria

**Keywords:** Agronomy, Genetic improvement, *Oryza sativa* L., Sustainability, Yield

## Abstract

**Context:**

With increasing labor shortage and production costs, water scarcity and climate change, there is increased interest in ratooning as a green, resource-efficient technology to boost sustainable rice production, especially in China. Since the performance of ratoon rice (regenerating a second crop from the stubble left in the fields after the main harvest) and the impact of agronomic practices on its yield have shown mixed results across the world, a better understanding is needed to determine under which conditions ratoon rice performs well.

**Objective:**

The objectives are (i) to quantify variation in rice yield of main and ratoon crops, (ii) to assess genetic variation in and impact of agronomic practices on rice yield, focusing on the yield of ratoon crop and total yield (main and ratoon crops), and (iii) review of economic and environmental benefits of ratoon rice in comparison with single and double rice cropping.

**Methods:**

In researching ratoon rice, we compiled a database from 68 studies published from 2000 to 2023. Descriptive data analysis was performed.

**Results:**

Studies from non-tropical regions account for about 70%. Large variation exists in the yield of ratoon crop across the studies, with lower yield from the tropics than non-tropics. The ratio of yield of ratoon crop to that of main crop also varied widely from 0.13 to 0.67 with 0.36 and 0.5 in tropics and non-tropics, respectively. The yield of ratoon crop was positively related to the yield of main crop, crop duration and nitrogen fertilizer application rate, which were generally higher in non-tropics. Hybrid varieties out-yielded inbred varieties in both main and ratoon crops in non-tropical regions. Direct seeding and AWD had a positive impact on the yield of ratoon crop. The impact of stubble cutting height was mixed. While agronomic nitrogen use efficiency (AEN) during entire ratoon rice cropping was similar to that reported for single rice cropping in previous studies, AEN for ratoon crop in tropical regions tended to be lower than those from previous studies on single rice cropping. Ratoon rice cropping reduced labor input and production cost and increased net economic return compared with double rice cropping.

**Conclusions:**

We propose a research agenda, with the focus on improvement of genetic and agronomic practices to explore the potential of ratoon rice cropping, especially in the tropics.

**Implications:**

This study provides insight into the progress in ratoon rice research over the past two decades globally, and specifically in the tropics.

## Highlights

• Ratoon rice cropping has recently received attention in non-tropical conditions.
• Data on ratoon rice cropping from 68 studies were compiled and analyzed.
• Large genetic exists in yield of ratoon crop with low yield in the tropics.
• Yield of ratoon crop was related to that of main crop and nitrogen application.
• We propose research on genetic improvement and improving agronomy in the tropics.

## 1 Introduction

Ratoon rice (*Oryza sativa* L.) is one which regenerates as a second crop from the stubble left in the fields after harvest of the main crop (IRRI, 1988). This technology is not new, but its adoption has been limited to a few countries such as China and USA, due to low yield, poor ratooning ability, and uneven maturity (IRRI, 1988). In USA, ratoon rice has been practiced since the 1960 s (Harrell et al., 2009). With release of short duration rice varieties, ratoon rice became popular in southern USA, where double-cropping rice is not possible owing to low temperatures. In 2020, more than 40% of the southwest Louisiana rice area was ratooned – nearly double that of 2000. An increase in the percentage of area producing a ratoon crop and the improvements of agronomic practices for ratoon rice contributed to yield growth rate (Kennedy et al., 2022). In China, with labor shortage and increasing costs together with research on agronomic practices for intensive ratoon rice cropping and the introduction of mechanical harvesting from 2010, ratoon rice became an attractive option for farmers (Peng et al., 2023), as it means savings in labor and less use of fertilizer, seeds, water and pesticides compared with double-cropping rice. The current total area of ratoon rice reached one million ha in China (Yu et al., 2022). Researchers in Japan also reported high rice yield with the ratoon rice system, equivalent to threefold the average yield achieved previously (Nakano et al., 2020). With water scarcity and climate change, an interest in ratooning as a green, resource-efficient technology is increasing, to provide a sustainable rice production system with reduced environmental impact (Yuan et al., 2019).

Rice is commonly ratooned only once, but there is a possibility that ratooning could be extended, especially with perennial rice (Zhang et al., 2023). Following successful hybridization between Asian rice [*O. sativa* (L.)] and its perennial African relative *Oryza longistaminata*, perennial rice varieties have been developed with the long-term goal of stabilizing production of rain-fed rice-based systems (Sacks et al., 2006). A recent study showed that from a single planting, an irrigated lowland rice system with perennial rice produced grains for eight consecutive harvests over four years, averaging 6.8 t/ha/harvest vs. the 6.7 t/ha/harvest of replanted annual rice in southern China (Zhang et al., 2023). By contrast, in Laos, where rice was grown under more severe environmental stress, there was a significant yield reduction of perennial rice in the second year (Samson et al., 2018).

As with the results from testing perennial rice in southern China and Laos, research on ratoon rice showed mixed results across different countries in tropical regions (Wang et al., 2020). Some studies were promising (e.g., Adigbo et al., 2012), whereas others showed significant yield reduction in the second season (e.g. Lal et al., 2023). With good agronomic practices, ratoon rice could give a yield equivalent to 60% of that of the main crop (Wang et al., 2020). But there was large variation in this ratio of yield of ratoon crop to yield of main crop in other studies. A wide range of agronomic practices has been recently tested for improving ratoon rice yield. These included variety selection, nutrient management, land preparation, crop establishment, water management, and stubble cutting height. Their impact on ratoon yield varied across studies (Wang et al., 2020). For example, lower stubble cutting height resulted in both positive and negative impacts on yield, (e.g. Nakano et al., 2020; Yang et al., 2022).

**Fig. 1:**
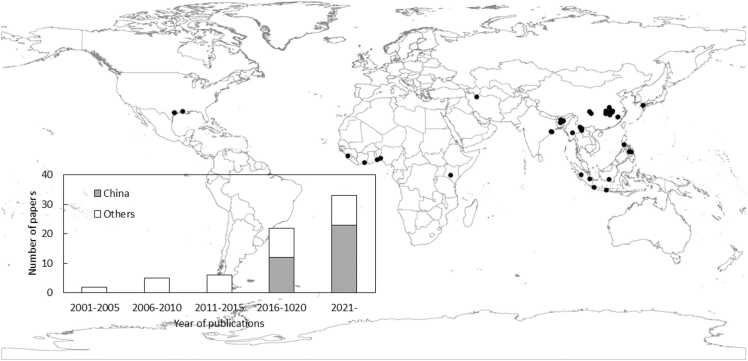
Locations of studies included in this review, and trends in the number of papers on ratoon rice included in this study.

Because the performance of ratoon rice including perennial rice and the impact of agronomic practices on yield have shown mixed results across the world, a better understanding is needed to determine under which conditions ratoon rice performs well. Research progress on rice ratooning over the past decades was comprehensively summarized by Wang et al. (2020). However, the review did not perform data analyses across the past studies to quantify the variation in yield of ratoon rice and the impact of agronomic practices. Also, after publication of Wang et al. (2020), there was a rapid increase in the number of papers in the past two years (Peng et al., 2023; also see Fig. 1).

Therefore, our objectives are (i) to quantify variation in rice yield of main and ratoon crops, (ii) to assess impact of agronomic practices on rice yield focusing on the yield of ratoon crop and total yield (main and ratoon crops), and (iii) review of economic and environmental benefits of ratoon rice in comparison with single and double rice cropping.

## 2 Materials and methods

A literature search was conducted on Scopus and GoogleScholar for articles published from 2000. In Scopus, we conducted search with ‘rice’ and ‘ratoon’, and found 206 papers. We used the same key words in GoogleScholar.

Only publications having data on yield of main and ratoon crops or total yield from field experiments and concrete tank experiment (1 study only – Shiraki et al., 2020) were selected. Here, we refer to ratoon rice as one that generates a second crop from the stubble left in the fields after harvesting the main crop. We include data from publications from perennial rice for planting and the first ratooning season. However, if perennial rice was harvested once a year only, this was not considered (e.g. Samson et al., 2018). We did not include publications using SALIBU technology, which was invented in Indonesia and refers to the harvest of ratoon crops 3.5–4 times annually and has been reported to realize an equivalent or a higher yield in ratoon crop than the main crop (Fitri et al., 2019), as there was not enough evidence to support this (e.g., Yamaoka and Ofori, 2020). If data from the same experiments were reported in multiple publications, a paper having data with the most complete dataset was used. Publications reporting in English in both abstract and main document were considered. With the above sampling and selection criteria, we compiled a database from 68 studies (Table 1).

**Table 1:** Overview of the studies used for analysis.

	Country	Province	PS^1^	Variety	Fertilizer	Land preparation	Crop establishment	Water	Cutting	Cropping system	Reference
1	USA		IL	✓ (7^2^)							Kiniry et al. (2001)
2	USA		IL	✓ (4)	✓						Bond and Bllich (2006)
3	USA		IL	✓ (3)							Bond and Bllich (2007)
4	USA		IL	✓ (6)							Dou et al. (2016)
5	USA		IL	✓ (40)							Cai et al. (2019)
6	USA		IL	✓ (4)	✓						Wang et al. (2021)
7	Benin		L	✓ (20)							Sanni et al. (2009)
8	Cote d’Ivoire		IL		✓						Kouakou et al. (2014)
9	Kenya		RU	✓ (5)							Ringera et al. (2014)
10	Nigeria		RL	✓ (11)							Adigbo et al. (2012)
11	Sierra Leone		RL	✓ (30)							Sanna et al. (2018)
12	Bangladesh		L		✓				✓		Begum et al. (2002)
13	Bangladesh		IL		✓						Shamiul Islam et al. (2008)
14	China	Fujian	IL		✓						Huang J. et al. (2022b)
15	China	Fujian	IL		✓						Weng et al. (2023)
16	China^3^	Henan	IL								Liu et al. (2016)
17	China	Henan	IL	✓ (8)							Zhang et al. (2021)
18	China	Henan	IL		✓						Zhang et al. (2022a)
19	China	Henan	IL	✓ (2)				✓			Zhang et al. (2022b)
20	China	Hubei	IL	✓ (2)							Zheng et al. (2022)
21	China	Hubei	IL	✓ (10)							Chen et al. (2018)
22	China	Hubei	IL	✓ (2)	✓						Wang et al. (2019)
23	China	Hubei	IL								He et al. (2019)
24	China	Hubei	IL		✓						Yang et al. (2021)
25	China	Hubei	IL		✓			✓			Ding et al. (2022)
26	China	Hubei	IL	✓ (2)			✓				Li et al. (2022)
27	China	Hubei	IL			✓					Jiang et al. (2022)
28	China	Hubei	IL							✓	Huang J. et al. (2022a)
29	China	Hubei	IL			✓					Li et al. (2022)
30	China	Hubei	IL	✓ (3)							Xia et al. (2022)
31	China	Hubei	IL					✓	✓		Yang et al. (2022)
32	China	Hubei	IL		✓				✓		Yu et al. (2022)
33	China	Hubei	IL							✓	Zhou et al. (2022)
34	China	Hubei	IL	✓ (2)							Zheng et al. (2023)
35	China	Hunan	IL			✓					Du et al. (2019)
36	China	Hunan	IL			✓	✓				Asenso et al. (2019)
37	China	Hunan	IL		✓						Zheng et al. (2019)
38	China	Hunan	IL	✓ (2)						✓	Xu et al. (2022)
39	China	Hunan	IL							✓	Zhang et al. (2022c)
40	China	Sichuan	IL		✓			✓			Zhang et al. (2019)
41	China	Sichuan	IL							✓	Song et al. (2020)
42	China	Sichuan	IL	✓ (4)		✓	✓				Jiang et al. (2021)
43	China	Sichuan	IL							✓	Song et al. (2021)
44	China	Sichuan	IL	✓ (5)							Song et al. (2022)
45	China	Yunnan	IL	✓ (22)							Zhang et al. (2017)
46	China	Yunnan	IL								Huang et al. (2018)
47	China	Yunnan	IL		✓		✓			✓	Zhang et al. (2021)
48	China	Yunnan	IL							✓	Zhang et al. (2023)
49	India		IL	✓ (6)						✓	Munda et al. (2009)
50	India		IL	✓ (2)			✓				Dwibedi et al. (2017)
51	India		L	✓ (4)							Kumar et al. (2021)
52	India		IL								Satapathy et al. (2022)
53	India		IL	✓ (10)	✓		✓		✓		Lal et al. (2023)
54	Indonesia		L	✓ (10)							Sinaga et al. (2014)
55	Indonesia		IL					✓	✓		Setiawan et al. (2014)
56	Indonesia		IL				✓				Pasaribu et al. (2018)
57	Indonesia		L	✓ (5)	✓						Gribaldi et al. (2020)
58	Indonesia		IL	✓ (15)							Zarwazi et al.(2021)
59	Iran		IL	✓ (4)			✓				Akhgari et al. (2013)
60	Japan		IL						✓		Nakano et al. (2020)
61	Japan		IL	✓ (2)							Nakano et al. (2021)
62	Japan		IL	✓ (2)							Tanaka et al. (2021)
63	Japan		IL	✓ (2)					✓		Nakano et al. (2022)
64	Myanmar		IL								Shiraki et al. (2021)
65	Myanmar		IL					✓			Shiraki et al. (2020)
66	Philippines		IL	✓ (2)							Torres et al. (2020)
67	Philippines		IL		✓						Banoc and Asio (2019)
68	Philippines		IL	✓ (6)					✓		Banoc et al. (2022)

1 IL: irrigated lowland; RL: rain-fed lowland; RU: rain-fed upland; and L: lowland (irrigated/rain-fed is not clearly indicated).

2 no. in parentheses indicates the number of varieties tested.

3 some studies do not have research topics belonging to listed categories.

Apart from recording data on yield, we also recorded the country name, the provincial name in the case of China, the rice production system (i.e. irrigated lowland, rainfed lowland, rain-fed upland), variety name and type (hybrid or inbred), crop duration, crop establishment, water management, inorganic nitrogen (N) application rate, stubble cutting height, and other agronomic practices. We did not consider phosphorus and potassium as most of the studies applied fertilizer containing these elements and most of studies dealing with inorganic fertilizer focused on nitrogen. Crop duration of the main crop is from sowing to harvest, whereas the duration of ratoon crop is from harvest of main crop (i.e. stubble cutting) to harvest of ratoon crop. Means for variables including yield in the given treatment were retrieved from each study. If data was shown in different years and locations, this was reported separately. In cases where data was only presented in figures, values were manually estimated.

We identified six popular agronomic practices tested in ratoon rice systems: variety, inorganic fertilizer management, land preparation, crop establishment, water management, and stubble cutting. Varieties tested in any given study range from one to 40. We selected papers including eight or more varieties for computing means and coefficient of variation (CV) in each study, performing correlation analysis to assess variation in yield of main and ratoon crops and to identify if yield of ratoon crop is related to that of main crop and total yield. We also compared yields between inbred and hybrid varieties. In each study, data from different treatments on agronomic practices was averaged for inbred and hybrid over years. If studies used more than one variety for each type, varieties were averaged for each type. For inorganic fertilizer management, we focus on papers having more than two N fertilizer application rates in total and used the polynomial trend to investigate relationships between N application rate and total rice yield across different studies. In each study, data for each N application rate was averaged across varieties over years. For inorganic N application rate, we considered its application of ten days and more after heading of the main crop as the same for the ratoon rice crop. Agronomic N use efficiency (AEN) was calculated as in the following equation for six studies which had included a N omission treatment.

AEN = [yield with N (kg/ha) – yield at zero N (kg/ha)]/N rate (kg N/ha) (1)

For land preparation, crop establishment, and water management, we categorized no-tillage and tillage, direct seeding and transplanting, and alternate wetting and drying (AWD) and continuous flooding, respectively. Then, we selected papers having such pair-comparison. No tillage and direct seeding were selected as they could reduce labor input in addition to labor-saving with ratoon rice cropping. Introduction of AWD could also help further reduction of water use. We considered tillage, transplanting, continuous flooding as control, and absolute difference in yield from control in each practice was reported. Data for each treatment was averaged across varieties over years. Different studies used different stubble cutting height. Thus, we visualized relationships between cutting height and ratoon yield in different studies using a scatter graph. Data was analyzed and visualized using Excel.

For comparing rice cropping patterns, we identified three papers, which enabled us to assess the impact of shift from annual double rice cropping and single cropping on economic and environmental sustainability. We considered yield, production costs, net economic return, net ecosystem economic benefit (NEEB), N application rate, pesticide use (kg/ha), labor (hrs/ha), and water use (m^3^). NEEB is usually used to assess the comprehensive economic benefit of crop production based on the benefit of crop yield and the cost of agricultural inputs and carbon footprint (Xu et al., 2022). As there were few papers dealing with this assessment, we included one paper conducting an on-farm survey to assess the economic and environmental benefits of different rice cropping patterns (Yuan et al., 2019).

## 3 Results

We compiled a database from 68 studies conducted in 14 countries, containing 750 observations on rice yield (Fig. 1). Here, we consider China, Japan, and USA as non-tropical regions, while others belong to tropical regions (Yuan et al., 2021). China had the highest number of publications, and accounted for around 50%, followed by USA, India and Indonesia (Table 1). The majority of papers were published from 2016 onwards. Except for one, all the studies were conducted in irrigated or rain-fed lowland rice conditions (Table 1). Most popular research topics were varietal evaluation, followed by inorganic fertilizer management.

### 3.1 Rice yield of main and ratoon crops

**Fig. 2:**
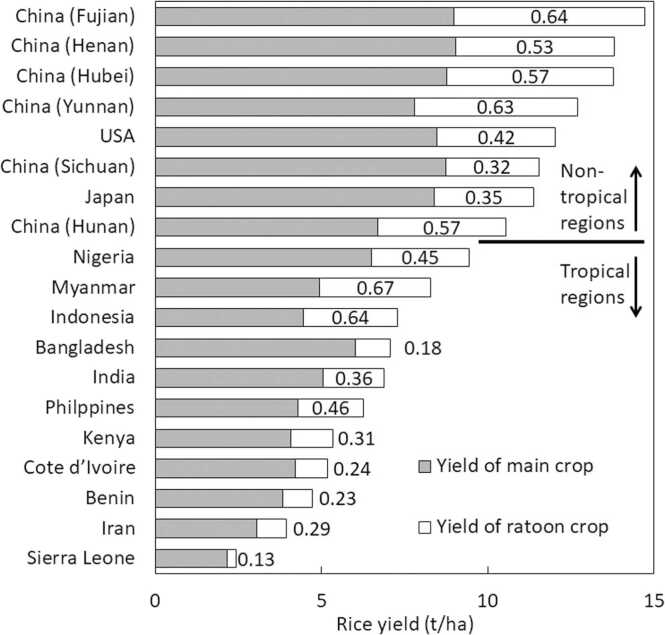
Rice yield of main and ratoon crops listed by country, and province for China, using data from studies listed in Table 1. Values in bar indicates relative yield of ratoon crop to that of main crop.

**Fig. 3:**
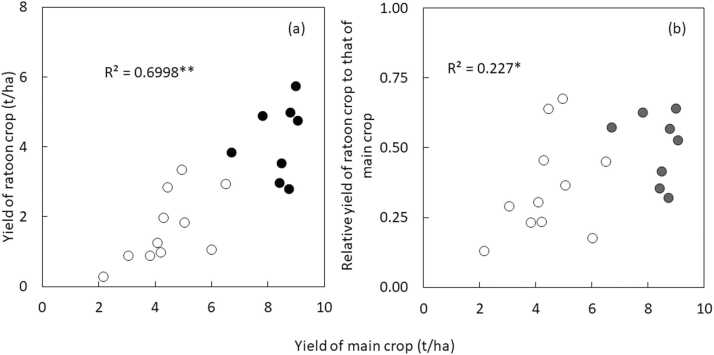
Relationship between rice yield of main crop and (a) yield of ratoon crop and (b) relative yield of ratoon crop to that of main crop. Data are from Fig. 2. White circles indicate data from tropical regions, while black circles indicate data from non-tropical regions. *Significance of the F-test at P = 0.05.**Significance of the F-test at P = 0.01.

Across all observations, the yield of main and ratoon crops ranged from 0.7 to 12.9 t/ha and from 0 to 8.9 t/ha, respectively, with a mean of 7.1 and 3.2 t/ha. The total yield ranged from 0.9 to 19.3 t/ha with an average of 10.3 t/ha. When data was disaggregated by country, and province for China, countries from non-tropical regions had a main crop yield of >8 t/ha with >3 t/ha of ratoon yield (Fig. 2), except for one province from China. In tropical regions, the yield of main and ratoon crops ranged from 2.2 to 6.5 t/ha and from 0.3 to 3.3 t/ha, respectively. The ratio of yield of ratoon crop to that of main crop also varied widely and ranged from 0.13 to 0.67 with on average 0.5 and 0.36 in non-tropical and tropical regions, respectively. When yields of main and ratoon crops were compared between Asian and African countries in tropical regions in Fig. 2, on average over countries, the yield of main and ratoon crops was higher in Asian countries with 4.6 vs. 4.2 t/ha and 2.0 vs. 1.3 t/ha, respectively. Furthermore, the ratio of yield of ratoon crop to that of main crop was higher in Asian countries (0.43 vs. 0.27). The rice yield of main crop was positively related to both yield of ratoon crop and ratio of yield of ratoon crop to that of main crop (Fig. 3).

**Fig. 4:**
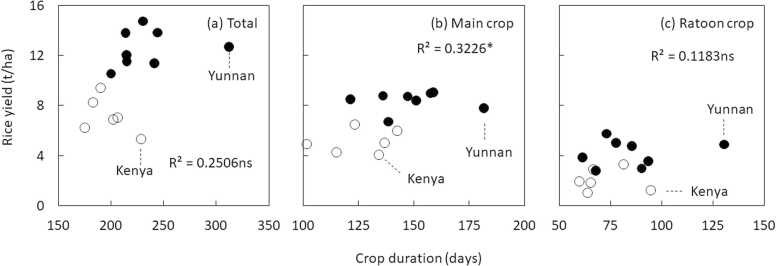
Relationship between crop duration and (a) total yield, (b) yield of main crop, and (c) yield of ratoon crop (n=12). As some countries did not have data on crop duration, sampling size is different from Fig. 2. White circles indicate data from tropical regions, while black circles indicate data from non-tropical regions. ns, non-significant. *Significance of the F-test at P = 0.05.

**Fig. 5:**
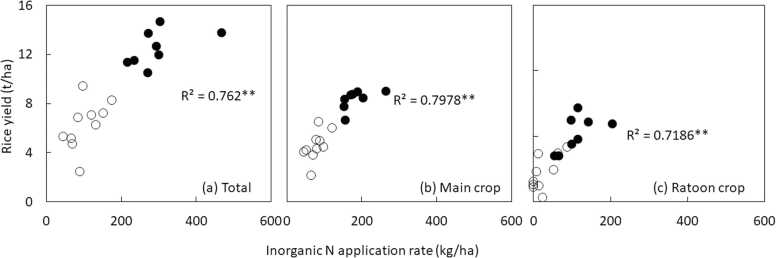
Relationship between inorganic N fertilizer application rate and (a) total yield, (b) yield of main crop, and (c) yield of ratoon crop (n=19; data from Iran is not included due to data unavailability). White circles indicate data from tropical regions, while black circles indicate data from non-tropical regions. **Significance of the F-test at P = 0.01.

We investigated the relationship between rice yield, crop duration and inorganic N fertilizer application rate using yield data shown in Fig. 2. We assumed that higher yield could be associated with longer duration and higher N application rate. The mean crop duration of main and ratoon crops was 139 and 78 days, respectively. Thus, ratoon crop had on average 61 days of shorter crop duration than main crop. Mean N application rate was 151 and 74 kg N/ha in main and ratoon crops, respectively. Both crop duration and N application rate had large variation across countries in both non-tropical and tropical regions (Fig. 4 and 5). On average, crop duration was 86, 68, and 81 in non-tropical regions, and Asian and African countries in tropical region, respectively. Similarly, total N application rate was 294, 132, and 73 kg N/ha non-tropical regions, and Asian and African countries. Thus, lower yield of ratoon crop in African countries is associated with N application rate rather than crop duration in tropical regions. Furthermore, correlation analysis revealed that the relationship between crop duration and total yield and yield of ratoon crop was positive, but not statistically significant (Fig. 4). This could be partially due to very long duration of ratoon rice in perennial rice in Yunnan and low yield of rainfed upland rice in Kenya (Fig. 4). When these data points were removed, the relationship between crop duration and total yield was highly significant (n=10, r=0.76, p<0.01). Higher rice yield of main and ratoon crops and total yield were associated with higher N application rate (Fig. 5).

**Fig. 6:**
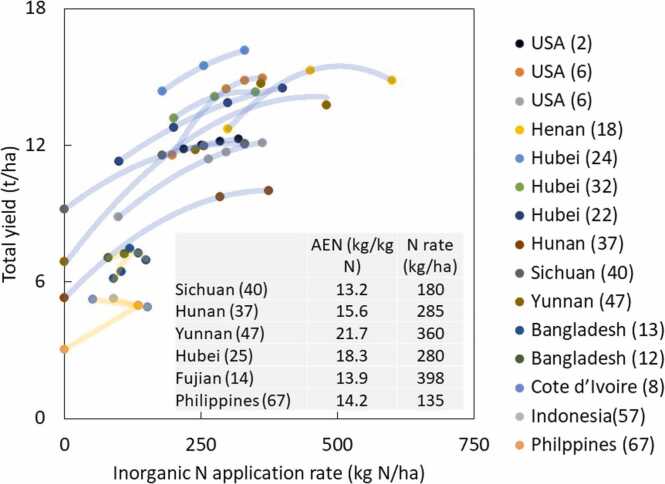
Relationship between inorganic N fertilizer application rate and total yield, and agronomic nitrogen use efficiency (AEN) at the given N application rate, which provided highest AEN among the treatments in the given study. Number after location name refers to # paper in Table 1. Polynormal regression was applied for each study. USA (6) had two data points as they had two experiments.

### 3.2 Varietal difference in rice yield of ratoon crop

We identified eight studies evaluating yield performance of ratoon rice using eight or more varieties. The trials were conducted in a wide range of conditions including high-yielding environments in non-tropical regions (i.e., China, USA), and low-yielding conditions (i.e. Indonesia, Sierra Leone). Consequently, a wide range of yield levels was observed in this list (Table 2). The CV of yield was generally higher in ratoon rice than in main rice with an average of 32 and 14%, respectively. In non-tropical regions especially, the CV of yield was small in main crop (3–10%). As we observed an apparent positive relationship between yield of main and ratoon crops in Fig. 3, we expected that there was a positive relationships between them in Fig. 4. However, except for a study from India, this was not the case, which indicated that high-yielding varieties in main crop were not always high-yielders in ratoon rice. Furthermore, the yield of ratoon crop had a greater association with total yield than the yield of main crop in non-tropical regions, whereas there was an opposite trend in tropical regions, except for India. Hybrid varieties out-yielded inbred varieties in main and ratoon, as well as total rice cropping in all cases except for one case for main crop (Table 3). An average yield gain with hybrid was 10, 29 and 16% in main and ratoon crops, and total rice cropping, respectively.

**Table 2:** Descriptive statistics for ratoon rice varietal trials evaluating yield performance of eight or more varieties in the studies listed in Table 1, correlation coefficients between yield of main and ratoon crops, and between them and total yield.

	Cropping pattern^1^	Mean yield (t/ha)	Relative yield of ratoon crop to that of main crop	Range in yield (t/ha)	CV of yield (%)	Correlation coefficient	
						Yield of main and ratoon crops	With total yield
USA (5^2^)	MR	9.0		7.3–9.6	7.6	0.39*^3^	0.75**
	RR	3.9	0.43	2.4–5.7	25.8		0.90**
Henan, China (17)	MR	8.8		8.3–9.0	2.8	0.29 ns	0.52 ns
	RR	5.3	0.60	4.1–6.2	15.8		0.97**
Hubei, China (21)	MR	8.8		6.4–11.2	10.4	-0.31 ns	0.49 ns
	RR	4.0	0.45	2.2–7.0	28.8		0.93**
Nigeria (10)	MR	6.7		4.8–8.1	16.1	-0.15 ns	0.77**
	RR	3.1	0.47	1.4–3.9	24.1		0.51 ns
India (53)	MR	5.3		4.1–6.3	12.7	0.94**	0.99**
	RR	1.9	0.37	0.7–2.7	31.4		0.98**
Benin (7)	MR	3.8		1.8–5.4	23.8	0.24 ns	0.96**
	RR	0.9	0.23	0.3–1.6	33.0		0.50*
Indonesia (54)	MR	2.6		2.1–3.5	15.6	-0.13 ns	0.68*
	RR	1.2	0.45	0.5–1.6	33.1		0.64*
Sierra Leone (11)	MR	2.2		0.7–3.3	26.0	0.45**	0.97**
	RR	0.3	0.13	0.1–0.9	63.5		0.66**

ns, non-significant. *Significance of the F-test at P = 0.05. **Significance of the F-test at P = 0.01.

1 MR: main crop; RR: ratoon crop.

2 refers to # paper in Table 1.

3 no. of varieties tested is shown in Table 1.

**Table 3:** Yield of main and ratoon crops between inbred and hybrid varieties.

	Year of testing	Variety type	Yield of main crop (t/ha)	Yield of ratoon crop (t/ha)	Relative yield of ratoon crop to that of main crop	Total yield (t/ha)	List of varieties tested
Hubei (21^a^)	2016	Inbred	7.3	4.2	0.57	11.5	HHZ
		Hybrid	9.2	5.7	0.62	14.9	TYHZ
	2017	Inbred	8.9	3.1	0.35	11.9	HHZ, ZJZ17, ZZ35, ZX1
		Hybrid	9.1	4.4	0.49	13.5	TYHZ, HLY898, LY287, LY76
Hubei (22)	2016–2017	Inbred	8.5	4.1	0.48	12.6	HHZ
		Hybrid	8.3	5.3	0.63	13.6	LY6326
Hubei (26)	2016	Inbred	8.1	4.4	0.54	12.5	HHZ
		Hybrid	8.9	5.4	0.60	14.3	TYGZ
Sichuan (42)	2017–2020	Inbred	8.3	3.0	0.36	11.3	HHZ, JNSM
		Hybrid	9.7	3.5	0.36	13.2	NY103, RY1015
USA (4)	2008–2009	Inbred	9.2	3.0	0.33	12.2	Bowman, Cocodrie, Presidio
		Hybrid	10.3	3.7	0.35	14.0	Clearfield XL729, XL745, XL723
Mean		Inbred	8.4	3.6	0.43	12.0	
		Hybrid	9.3	4.7	0.50	13.9	
Relative yield gain with hybrid (%)			10	29	16	16	

a refers to # paper in Table 1.

### 3.3 Effect of inorganic N fertilizer application

**Fig. 7:**
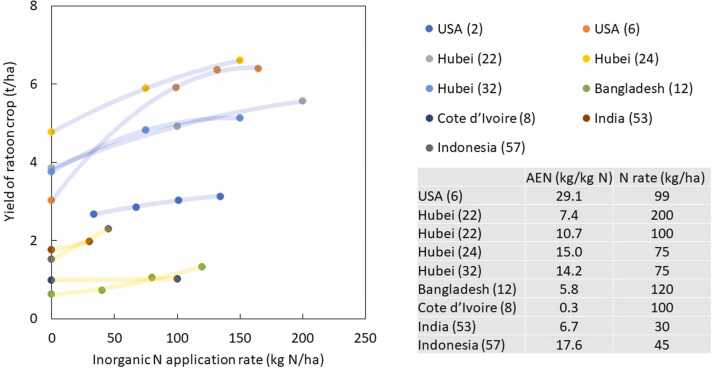
Relationship between inorganic N fertilizer application rate and rice yield of ratoon crop, and agronomic nitrogen use efficiency (AEN) at the given N application rate, which provided highest AEN among the treatments in the given study. Number after location name refers to # paper in Table 1. Polynormal regression was applied for each study. Hubei (22) had two data points for AEN, as they had two different fertilizer applications rates on the same experiments. One had only two N rates. Therefore, this one is not shown in the Figure.

Fig. 5 shows the relationships between inorganic N application rate and total yield across 14 studies. As data points from tropical regions was limited, we added two studies that had two fertilizer treatments from tropical regions (Ding et al., 2022; Huang J. et al., 2022b). This figure clearly shows that the studies from tropical regions had a lower fertilizer application rate as well as lower yield. When the N application rate was around 100 kg N/ha, total yield was between 8.8 and 11 t/ha in non-tropical regions except for Hunan, whereas the yield was up to 7.4 t/ha in tropical regions. Such a large difference was most likely due to a difference in soil indigenous nitrogen supply, for which rice yield from no N application has been used as proxy (Dobermann et al., 2003). The yield at no N application was lower in Hunan, China and Philippines than in others. Agronomic nitrogen efficiency (AEN) was calculated in six studies (Fig. 5), and ranged from 13.2 to 21.7 kg/kg N. These values of AEN were the highest among the fertilizer treatments in each study. For these AENs, the N fertilizer application rate ranged from 135 to 398 kg/kg N. AEN from Philippines was similar to those from Sichuan and Fujian. Fig. 7 shows the effect of inorganic N application rate on ratoon rice across nine studies, which had the same level of inorganic N application as main crop. Studies in non-tropical regions had a higher yield than in tropical regions. As inorganic N fertilizer was applied at main crop, residual N fertilizer might have also contributed to higher yield in non-tropical regions. Three studies from non-tropical regions and one from tropical showed comparable to or higher AEN than those observed for total yield in Fig. 7. Studies from Bangladesh, Cote d’Ivoire, and India had low AEN, despite their low yield at zero N.

### 3.4 Effect of no-tillage, direct seeding, alternate wetting and drying, and stubble cutting height

**Fig. 8:**
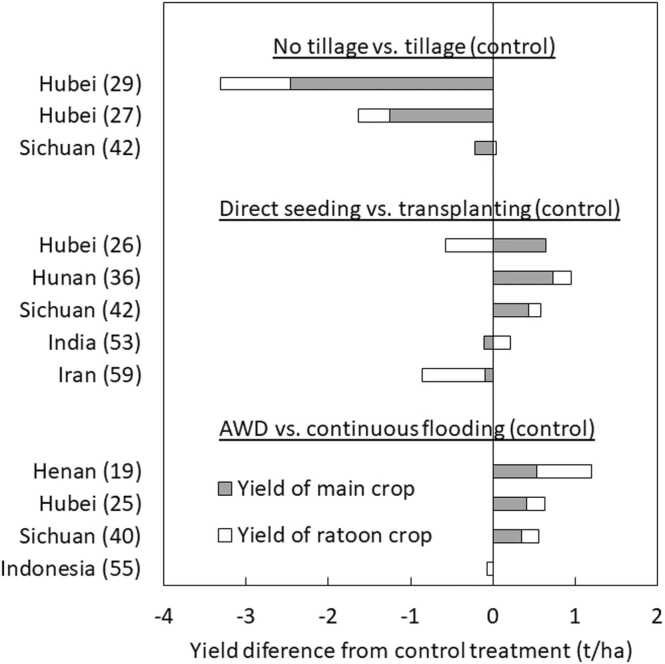
Effect of no tillage, direct seeding, and alternate wetting and drying on yield of main and ratoon crops. Number after location name refers to # paper in Table 1.

Among studies dealing with land preparation, crop establishment or water management, major practices tested as alternative options were no-tillage, direct seeding, and alternate wetting and drying (AWD). Each had three to five studies (Fig. 8). Unfortunately, these studies did not provide data on labor saving for no-tillage and direct seeding or water use and water productivity for all of them. No-tillage consistently reduced the rice yield of main crop and total yield across three studies. AWD had positive impact on yield of main and ratoon crops in all studies. Although direct seeding had a negative impact on rice yield of ratoon crop in two out of five studies, it had a positive impact on the total yield in all the countries except for Iran.

**Fig. 9:**
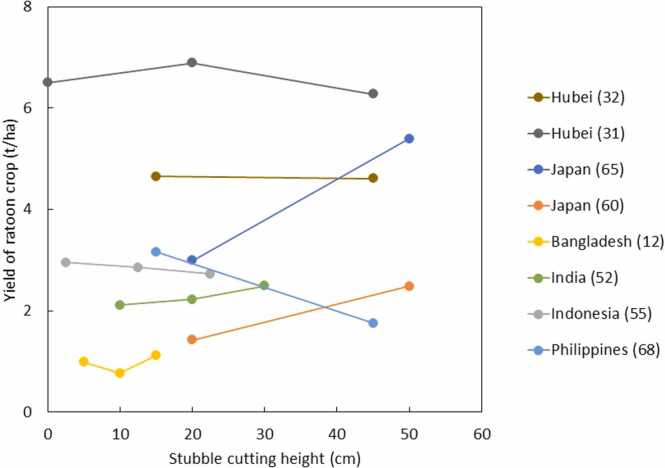
Effect of stubble cutting height on rice yield of ratoon crop. Number after location name refers to # paper in Table 1.

Studies dealing with stubble cutting height used a wide range of measurements, ranging from zero to 50 cm (Fig. 9). Except for two studies from Japan and one from the Philippines, there was no large difference in rice yield of ratoon crop across the treatments. Two studies from Japan reported that higher cutting has a positive effect, whereas the opposite result was observed in the Philippines.

### 3.5 Effect of shift from single or double rice cropping to rice ratoon cropping

Four studies showed the impact of change from double or single rice cropping (reefing as to DR or SR) to rice ratoon cropping (RR), on various economic and environmental indicators. We considered total yield, production costs, net economic benefit, and net ecosystem economic benefit (NEEB), plus labor input as economic indicators, with N fertilizer application rate, pesticide application rate, and water use as environmental indictors (Table 4). In comparison with DR, RR could reduce production costs by 21–40%, labor input by 32–45%, and increase net economic return by 55–104% or NEEB by 31–66%. Yield reduction ranged from one to 14% except for India, which had yield gain with RR. Result for N fertilizer application rate was not consistent across studies, whereas pesticide application and water use were reduced with RR. It is important to mention that RR could also reduce total crop duration in comparison with DR. This could allow farmers to have additional crops after RR, depending on weather after the ratoon crop. Shifting from SR to RR requires additional investment including fertilizer, labor input, and water use. However, with additional yield from ratoon rice, net economic return or NEEB could be increased. It is noted that this analysis does not include any non-crop after SR or RR.

**Table 4:** Effect of shift from double or single rice cropping to ratoon cropping on economic and environmental indicators (%).

	Hubei, China (28^a^)	Hubei, China (Yuan et al. 2019)	Hunan, China (38)	India (49)
**From double rice cropping to ratoon rice cropping**				
Total yield	-6	-14	-1	9
Production cost	-40	-40	-32	-21
Net economic return	NA	104	NA	55
Net ecosystem economic benefit	66	NA	31	NA
Labor input	-45	-32	NA	NA
N fertilizer application rate	8	-28	17	NA
Pesticide application rate	-45	-36	-12	NA
Water use	-41	-18	NA	NA
**From single rice cropping to ratoon rice cropping**				
Total yield	57	71	NA	84
Production cost	3	48	NA	32
Net economic return	NA	129	NA	166
Net ecosystem economic benefit	177	NA	NA	NA
Labor input	1	233	NA	NA
N fertilizer application rate	84	51	NA	NA
Pesticide application rate	-12	-9	NA	NA
Water use	14	36	NA	NA

a refer to # paper in Table 1.

## 4 Discussion

This study acknowledges that the dataset used in our study was geographically biased, with around 50% of studies coming from China and very few from Africa and none from Latin America, highlighting the paucity of data in the regions. We did not consider biophysical factors such as weather conditions and soil properties for assessing the performance of ratoon rice cropping. This is because varieties used and stubble cutting height varied. Recent studies indicated that the optimum cutting height might depend on temperature during the ratoon cropping period (Hu et al., 2023, Nakano et al., 2023). Furthermore, ratooning ability and yield of ratoon crop can be affected by the stubble height (Wang et al., 2020). The optimum stubble cutting height of the main crop varies, depending on the ratooning characteristics of the varieties (Harrell et al., 2009; Yao et al., 2022). With this complex interaction, more detailed studies are needed to quantify the impact of biophysical factors and their interaction with variety and stubble height cutting. We also acknowledge the small number of studies for assessing the impact of individual practices [from three (no-tillage, AWD) to 14 (N fertilizer application)]. Such a limited number does not allow us to conduct the comprehensive meta-analysis that has been carried out by other studies (Pittelkow et al., 2015, Carrijo et al., 2017, Chivenge et al., 2021). With increased research interest in ratoon rice and a greater number of publications (Table 1), we believe that in the near future a more rigorous evaluation of ratoon rice cropping will be possible. Nevertheless, this is the first study to quantify the variation in rice yield of main and ratoon crops and total yield, to review the field evaluation of selected agronomic practices, including rice varieties in ratoon rice cropping, in the past two decades and to assess the impact of a shift from double or single rice cropping to ratoon rice cropping on economic and environmental indicators. We discuss major findings in four sub-sections below.

### 4.1 Variation in yield in ratoon rice cropping and its association with factors

We have shown that large variation in yield of main and ratoon crops and total yield exists in ratoon rice cropping across the world, and the variation is related to the agro-ecological zone (tropical vs. non-tropical regions), inorganic N fertilizer application and crop duration (Fig. 2, Fig. 4, Fig. 5). A lower yield of ratoon crop and ratio of yield of ratoon crop to that of main crop in tropical regions is associated with a lower yield of main crop. Production systems could explain yield variations, but the limited number of studies from rain-fed lowland and upland rice prevents comparison. Our findings are consistent with findings by Yuan et al. (2021), which assessed yield gap and resource-use efficiency across 32 rice cropping systems with annual rice at global level. The study found that the yield gap of annual rice is smaller in non-tropical countries such China and USA, and is attributed to inorganic N fertilizer application, implying that reducing the yield gap in some countries in south-east Asia and Africa require increased N application. Enhancing yield and closing the yield gap of main crop could also contribute to increased yield of the ratoon crop as they are positively related (Fig. 3). Although crop duration can be increased through selection of varieties in tropical regions, such options might not be attractive to farmers as longer duration requires additional production costs such as labor and water and does not allow farmers to intensify land use in irrigated systems and increase risk for terminal water stress at the end of the wet season in rain-fed systems. Furthermore, a lower yield of ratoon crop in African countries is associated with low N application rate in tropical regions. Rather than solely relying on increased inorganic N application rate, improving N use efficiency is needed in African countries as proposed by Johnson et al. (2023), Peng et al. (2023), see N application method for China in 4.3), and Saito et al. (2023). Thus, research should focus on identifying high-yielding varieties and improving agronomic practices in ratoon rice cropping in tropical regions and demonstrate scientific evidence on the potential of intensive ratoon rice cropping there. We discuss this in the following sub-sections.

### 4.2 Genetic improvement

Up to now, there has been no specific breeding program for ratoon rice, except for perennial rice (Zhang et al., 2023). Thus, except for this case, the varieties used in ratoon rice cropping are selected from those that were developed for other purposes (Wang et al., 2020). This study shows that a large variation in rice yield of ratoon crop exists. Thus, exploring genetic variation in rice yield could enhance total rice productivity (Table 2). In China and USA, a smaller variation in the yield of main crop compared to ratoon yield observed could be because existing high-yielding varieties (e.g. hybrid) already achieved high yield potential (Sha et al., 2007, Zhang et al., 2009), resulting in low variation in main crop yield. Our yield comparison between inbred and hybrid varieties (Table 3) revealed consistent yield advantage of hybrids in both main and ratoon crops across the studies. This confirms previous efforts in China summarized by Peng et al. (2023). The use of hybrid varieties in ratoon rice cropping seems more economic than in double or single rice cropping, as yield gain with hybrids is similar between main and ratoon crops (0.9 vs. 1.0 t/ha), and ratoon rice cropping can save the cost of hybrid seed for one season.

Although the yield of ratoon crop was positively related to that of main crop when comparison was made across studies (Fig. 3), there were no such positive relationship in varietal evaluation except for a few cases in Table 2 and yield comparison between inbred and hybrid varieties. No or poor relationships between the yield of main and ratoon crops might be attributed to genetic variation in ratooning ability, which refers to dormant buds being able to regenerate from the nodes of the stubble; this is fundamental to the yield formation of ratoon crop (Wang et al., 2019). Ratooning ability was defined as the ratio of panicle number/m^2^ in the ratoon crop to that in the main season (Wang et al., 2020). Ratooning ability can be also affected by the stubble height (Wang et al., 2020). The optimum stubble cutting height of the main crop varies, depending on the ratooning characteristics of the varieties (Harrell et al., 2009; Fan et al., 2022). Some varieties have a strong ability to regenerate from the basal nodes of the stubble, whereas others mainly rely on panicles regenerated from the upper nodes. This difference in ratooning characteristics could be the main reason we had mixed results from stubble cutting height (Fig. 8). It has been considered that crop duration of ratoon crop is also regulated by stubble cutting and with low cutting height, crop duration can be extended (Wang et al., 2020). Yang et al. (2022) supported this theory, whereas studies from Japan and India had the opposite result (Nakano et al., 2022; Lal et al., 2023). This could be also attributed to the varietal difference in adaptation to cutting height. Recent studies indicated the optimum cutting height might depend on temperature during the ratoon cropping period (Hu et al., 2023, Nakano et al., 2023). These suggest that further research is needed for identifying a mechanism for genotype by stubble height cutting by environmental interaction for ratooning availability and yield, identifying a mechanism conferring a high-yielding ability of hybrid varieties in a ratoon crop, and identifying varieties having superior ratooning availability for the given cutting height. The first step could be to identify target cutting height based on local farmers’ practices and existing equipment for harvesting. If farmers already used mechanized harvesting, target-cutting height should be based on this equipment. Otherwise, adopting more new varieties targeting different cutting height would require advanced machinery for harvesting. In addition, research also needs to consider water conditions before and after harvesting of the main crop. These conditions were not often well described in detail in most publications. Shiraki et al. (2020) reported that yield in the dry regime during this period was higher by 69% than that in the saturated regime, and indicated that soil oxidation conditions during the initial growth period of ratoons could contribute to the improved yield of ratoon crops. Furthermore, soil drying also mitigates yield loss in the ratoon crop due to damage by crushing of the stubble remaining after mechanical harvesting of the main crop (Zheng et al., 2022). Thus, for varietal screening, it is important that varieties having similar crop duration can be grown together so that water conditions are the same before harvesting across the varieties to be tested. Otherwise, ratooning ability observed in varietal screening can be masked by a difference in water conditions.

Increasing crop duration through genetic improvement is another important trait for maximizing total yield. However, this can depend on the local context. Water availability and temperature can determine the maximum growing period for ratoon rice cropping. The use of a crop simulation model adapted to ratoon rice cropping can be useful for identifying the optimum sowing time and crop duration to be targeted through breeding (Ling et al., 2019).

### 4.3 Agronomy

The negative impact of no-tillage on rice yield in this review confirms results from meta-analysis, comparing conventional tillage to no-till practices for rice (Pittelkow et al., 2015), which revealed that no-tillage reduced the yield of annual rice on average by 7.5%. As one out of three reported that no-tillage and tillage had similar yield levels (Jiang et al., 2021), no-tillage might be suitable for the specific conditions. As a previous study could not determine under what conditions no-tillage performed well for rice (Pittelkow et al., 2015), further studies are needed to identify target domains for no-tillage.

Direct seeding is well known as an alternative option for transplanting, which can potentially reduce the consumption of resources and emission of greenhouse gases (Kumar and Ladha, 2011). This review showed a positive impact of direct seeding on total yield in four out of five studies, and two studies had a negative impact on yield of ratoon crop. However, one of the studies used two varieties. One variety – hybrid – had the same yield of ratoon crop between direct seeding and transplanting (Dong et al., 2017). Thus, the use of varieties adapted to ratoon rice cropping could minimize potential risk of yield reduction in ratoon crop, when direct seeding is introduced.

Our review for consistent results of the impact of AWD on the yield of main and ratoon crops confirms previous studies indicating that soil drying is important for ratoon crops (Hasegawa and Yoshida, 1982), and continuous flooding reduces the growth of the ratoon crop by decreasing the growth of roots and the buds from the basal nodes (Bahar and De Datta, 1977). Our observation is also supported to some extent by Carrijo et al. (2017) who conducted a meta-analysis for assessing the impact of AWD, and found that the yield of annual rice was not reduced when a mild level of soil drying was imposed. Our observation was also supported by another study (Shiraki et al., 2020; see above discussion in 4.1). Such water management is only possible if farmers can manage water themselves. Where irrigation authorities are responsible for the distribution of water, collective action is needed for ratoon rice cultivation under AWD. Even in rain-fed systems, supplemental irrigation and a proper drainage system might be key to making sure that the ratoon crop does not suffer serious water stress (Samson et al., 2018). Further research is needed to optimise soil moisture content depending on the production system (i.e. irrigated vs. rain-fed), land preparation (e.g. puddled vs. non-puddled), crop establishment method (e.g. direct seeding vs. transplanting) and ease of controlling water (e.g. irrigation and drainage).

Positive relationships between total yield, duration and N application rate in this study confirmed other studies on annual rice grown under irrigated conditions (Ying et al., 1998, Katsura et al., 2008). Total yield as well as yield of ratoon crop with no N fertilizer application, which is proxy for indigenous soil nitrogen supply, was low in tropical regions. As the number of studies in tropical regions is limited in this review, further research is needed to quantify indigenous soil nitrogen supply for ratoon rice cropping in tropical regions. Here, we use data from a previous study from Asia to estimate the typical total yield without N application rate in irrigated rice. Dobermann et al. (2003) reported that the high-yielding season (i.e. dry season) and low-yielding season (i.e. wet season) had 4.4 and 3.5 t/ha of mean yield of annual rice grown without N fertilizer application under irrigated conditions across Asian countries. If a main rice crop is grown during the high-yielding season and a ratoon crop has 0.5 of relative yield to that of main crop (i.e., 4.4 ×0.5 = 2.2), which is equivalent to 63% of yield of annual rice in low-yielding season (i.e., 2.2/3.5 = 0.63), the total yield without N fertilizer application could become 6.6 t/ha. This yield is higher than Hunan, China in Fig. 6 and similar to Yunnan, China. Although this calculation needs to be validated through field experiments, indigenous soil nitrogen supply in tropical regions would not always be lower than non-tropical regions. Regarding a low yield of the ratoon crop, we did not consider N fertilizer application rate in the main crop. Non-tropical regions tended to receive higher N application rate than the tropical regions (100–200 vs. 52–90 kg/kg N), and produce higher yield (8.6–9.6 vs. 3.5–6.2 t/ha). Such difference might have resulted in a large difference in yield of ratoon crop without N fertilizer application between non-tropical and tropical regions. Increasing yield further without N fertilizer application requires better agronomic practices such as water management, stubble cutting height, and choice of variety as discussed above.

Agronomic N use efficiency (AEN) of the total cropping period (main and ratoon crops) is similar to that reported for annual rice in tropical regions (Dobermann et al., 2002, Chivenge et al., 2021), and results from meta-analysis in annual rice in China (Che et al., 2015). However, AEN tends to be lower for ratoon crop in tropical regions. Peng et al. (2023) showed unique fertilizer management practices recommended to apply bud-promoting N fertilizer two weeks before the harvesting of the main crop and tiller promoting N fertilizer within three days after harvest of the main crop. Water management as mentioned above for soil drying before and after harvesting as well as AWD during ratoon crop (Shiraki et al., 2020, Ding et al., 2022) could help to improve AEZ. Such practices were not yet introduced in tropical regions, and their testing is warranted for improving AEN for ratoon crop in tropical regions. In addition, a site-specific nutrient management (SSNM) approach (Chivenge et al., 2021, Chivenge et al., 2022) an a controlled-release N fertilizer can be introduced to ratoon rice cropping for improving AEN. With SSNM, the fertilizer nutrient requirements for a specific field are calculated from the difference between the total amount of nutrient required by the crop to achieve a given target yield and the nutrient supply, which reflects the amount of a particular nutrient (N, P or K) available from the soil, crop residues, irrigation water or biological N fixation during one crop cycle (Dobermann et al., 2002, Chivenge et al., 2021). The SSNM approach has been shown to increase yield, profitability and nutrient use efficiency of annual rice (Chivenge et al., 2021). It could avoid excessive N application and provide the correct nutrient balance for improving agronomic N use efficiency in ratoon rice cropping. We understand that controlled-release N fertilizer was tested in one study in China only (Ding et al., 2022). Further study is warranted not only for improving AEN, but also for reducing labor input in applying N fertilizer.

Our assessment of ratoon rice cropping in comparison with double or single cropping reveals its economic and environmental benefits (Table 4). As the number of studies is very few, this type of assessment should be conducted in tropical regions to calculate the potential benefit of intensive ratoon rice cropping. Since data on the economic and environmental benefits of emerging production systems such as perennial rice system and SALIBU technology is scarce, further research is needed. Future research should also take into account a cropping system beyond rice cropping. For example, ratoon rice cropping can be compared with rice in rotation with a non-rice crop as well.

## 5 Conclusions

Ratoon rice is not new technology and was extensively cultivated previously as a low-input cropping system. However, in China, due to increasing input and labor costs, farmers have adopted ratoon rice cropping, and cultivated it more intensively than before. Researchers in China have been encouraged to conduct research into intensifying ratoon rice cropping and have demonstrated its economic and environmental benefits, as shown in this study. By contrast with China, such scientific evidence is not yet available in tropical regions, which are facing labor shortage and increasing production costs (e.g., Devkota et al., 2019). This study has shown that there is a large gap in research into ratoon rice between China and tropical regions. Overall, improved agronomic practices in China has become more complicated compared with single or double rice cropping. In conclusion, we argue the need for feasibility research into the potential of intensive ratoon rice cropping in tropical regions as a first step towards the development of sustainable rice production systems. The proposed research agenda includes the following:

• Assessing the genetic variation in ratooning ability and yield of ratoon crops in existing inbred and hybrid varieties.
• Testing of intensive agronomic practices shown in this study to demonstrate the potential of ratoon rice cropping.
• Assessment of economic and environmental impacts of ratoon rice cropping in comparison with locally common rice cropping.
• Identifying target domains where ratoon rice cropping can perform well through GIS, remote-sensing, field survey, and crop simulation model.
• Assessment of farmers’ perception of ratoon rice cropping, and identifying barriers/enabling factors for its large-scale adoption.

These studies could also include perennial rice systems and SALIBU technology. The results from these studies could help determine whether further investment on research on ratoon rice cropping is necessary.

## CRediT authorship contribution statement

**Elliott Ronald Dossou-Yovo:** Conceptualization, Writing – review & editing. **Kazuki Saito:** Conceptualization, Data curation, Formal analysis, Methodology, Writing – original draft. **Ali Ibrahim:** Conceptualization, Writing – review & editing.

## Declaration of Competing Interest

The authors declare that they have no known competing financial interests or personal relationships that could have appeared to influence the work reported in this paper.

## Data Availability

Data will be made available on request.

## References

[bib1] Adigbo S.O., Olojede M.O., Harris P.J.C., Ajayi O. (2012). Ratooned lowland NERICA rice varieties as an option for triple cropping in inland valleys of derived savannah in Nigeria. Exp. Agric..

[bib2] Akhgari H., Noorhosseini-Niyaki S.A., Sadeghi S.M. (2013). Effects of planting methods on yield and yield components of ratoon and main plant of rice (*Oryza sativa* L.) in Rasht, Iran. Indian J. Fundam. Appl. Life Sci..

[bib3] Asenso E., Zhang L., Tang L., Issaka F., Tian K., Li J., Hu L. (2019). Moldboard plowing with direct seeding improves soil properties and sustainable productivity in ratoon rice farmland in southern China. Sustainability.

[bib4] Bahar F.A., De Datta S.K. (1977). Prospects of increasing tropical rice production through ratooning 1. Agron. J..

[bib5] Banoc D.M., Asio V.B. (2019). Response of lowland rice (*Oryza sativa* L.) to fertilization when grown as main and ratoon crop. *Annuals*. Trop. Res..

[bib6] Banoc D.M., Servillano R., Asio V.B. (2022). Ratooning response of lowland rice (*Oryza sativa* L.) varieties to cutting height of ratoon crop. SVU-Int. J. Agric. Sci..

[bib7] Begum, Hasan M.K., Hossain K.M., Hossain S.M.A., M.A (2002). Effect of culm cutting height and nitrogenous fertilizer on the yield of ratoon of late Boro rice. Pak. J. Agron..

[bib8] Bond J.A., Bollich P.K. (2006). Ratoon rice response to nitrogen fertilizer. Crop Manag..

[bib9] Bond J.A., Bollich P.K. (2007). Effects of pre-harvest desiccants on rice yield and quality. Online. Crop Prot..

[bib10] Cai H., Tabien R.E., Xu D., Harper C.L., Samford J., Yang Y., You A., Samonte S.O.P.B., Holgate L., Jiao C. (2019). Grain quality and yield of rice in the main and ratoon harvests in the southern U.S. J. Agric. Sci..

[bib11] Carrijo D.R., Lundy M.E., Linquist B.A. (2017). Rice yields and water use under alternate wetting and drying irrigation: a meta-analysis. Field Crops Res..

[bib12] Che S., Zhao G., Li Y., Yuan L., Li W., Lin Z., Hu S., Shen B. (2015). Review grain yield and nitrogen use efficiency in rice production regions in China. J. Integr. Agric..

[bib13] Chen Q., He A., Wang W., Peng S., Huang J., Cui K., Nie L. (2018). Comparisons of regeneration rate and yields performance between inbred and hybrid rice cultivars in a direct seeding rice-ratoon rice system in central China. Field Crops Res..

[bib14] Chivenge P., Saito K., Bunquin M.A., Sharma S., Dobermann A. (2021). Co-benefits of nutrient management tailored to smallholder agriculture. Glob. Food Secur..

[bib15] Chivenge P., Zingore S., Ezui K.S., Njoroge S., Bunquin M.A., Dobermann A., Saito K. (2022). Progress in research on site-specific nutrient management for smallholder farmers in sub-Saharan Africa. Field Crops Res..

[bib16] Devkota K.P., Pasuquin E., Elmido-Mabilangan A., Dikitanan R., Singleton G.R., Stuart A.M., Vithoonjit D., Vidiyangkura L., Pustika A.B., Afriani R., Listyowati C.L. (2019). Economic and environmental indicators of sustainable rice cultivation: A comparison across intensive irrigated rice cropping systems in six Asian countries. Ecol. Indic..

[bib17] Ding Z., Li J., Hu R., Xiao D., Huang F., Peng S., Huang J., Li C., Hou J., Tian Y., Zhou J., Cao B. (2022). Root-zone fertilization of controlled-release urea reduces nitrous oxide emissions and ammonia volatilization under two irrigation practices in a ratoon rice field. Field Crops Res..

[bib18] Dobermann A., Witt C., Dawe D., Abdulrachman S., Gines H.C., Nagarajan R., Satawathananont S., Son T.T., Tan P.S., Wang G.H., Chien N.V. (2002). Site-specific nutrient management for intensive rice cropping systems in Asia. Field Crops Res..

[bib19] Dobermann A., Witt C., Abdulrachman S., Gines H.C., Nagarajan R., Son T.T., Tan P.S., Wang G.H., Chien N.V., Thoa V.T.K., Phung C.V. (2003). Soil fertility and indigenous nutrient supply in irrigated rice domains of Asia. Agron. J..

[bib20] Dong H., Chen Q., Wang W., Peng S., Huang J., Cui K., Nie L. (2017). The growth and yield of a wet-seeded rice-ratoon rice system in central China. Field Crops Res..

[bib21] Dou F., Tarpley L., Chen K., Wright A.L., Mohammad A. (2016). Planting date and variety effects on rice main and ratoon crop production in south Texas. Commun. Soil Sci. Plant Anal..

[bib22] Du P., Luo H.W., He L.X., Mao T., Lai R.F., Tang X.R., Tang Q.Y., Hu L. (2019). The effect of plough tillage on productivity of ratooning rice system and soil organic matter. Appl. Ecol. Environ. Res..

[bib23] Dwibedi S.K., De G.C., Dhua S.R. (2017). Relative performance of rice (*Oryza sativa*)–ratoon production system as influenced by date of sowing and system of cultivation of plant rice genotypes. Indian J. Agronomy.

[bib24] Fitri R., Kusnadi N., Yamaoka K. (2019). SALIBU technology in Indonesia: an alternative for efficient use of agricultural resources to achieve sustainable food security. Paddy Water Environ..

[bib25] Gribaldi G., Nurlaili N., Sakalena F., Dewi N., Asroh A. (2020). Strategy of nitrogen fertilizer application to increase growth and yield of rice in ratoon system at tidal swampland. Aust. J. Crop Sci. 14.

[bib26] Harrell D.L., Bond J.A., Blanche S. (2009). Evaluation of main-crop stubble height on ratoon rice growth and development. Field Crops Res..

[bib27] Hasegawa S., Yoshida S. (1982). Water uptake by dryland rice root system during soil drying cycle. Soil Sci. Plant Nutr..

[bib28] He A., Wang W., Jiang G., Sun H., Jiang M., Man J., Cui K., Huang J., Peng S., Nie L. (2019). Source-sink regulation and its eﬀects on the regeneration ability of ratoon rice.. Field Crops Res..

[bib29] Hu X., Ma M., Huang Z., Wu Z., Su B., Wen Z., Fu Y., Pan J., Liu Y., Hu R., Li M. (2023). Progress and challenges of rice ratooning technology in Guangdong Province. China *Crop Environ.*.

[bib30] Huang G., Qin S., Zhang S., Cai X., Wu S., Dao J., Zhang J., Huang L., Harnpichitvitaya D., Wade L.J., Hu F. (2018). Performance, economics and potential Impact of perennial rice PR23 relative to annual rice cultivars at multiple locations in Yunnan province of China. Sustainability.

[bib31] Huang J., Yu X., Zhang Z., Peng S., Liu B., Tao x, He A., Deng N., Zhou Y., Cui K., Wang F., Huang J. (2022). Exploration of feasible rice-based crop rotation systems to coordinate productivity, resource use efficiency and carbon footprint in central China. Eur. J. Agron..

[bib32] Huang J., Wu J., Chen H., Zhang Z., Fang C., Shao C., Lin W., Weng P., Khan M.U., Lin W. (2022). Optimal management of nitrogen fertilizer in the main rice crop and its carrying-over effect on ratoon rice under mechanized cultivation in Southeast China. J. Integr. Agric..

[bib33] IRRI (1988). Rice ratooning. Los BañOs., Philipp..

[bib34] Jiang P., Xu F., Zhang L., Liu M., Xiong H., Guo X., Zhu Y., Zhou X. (2021). Impact of tillage and crop establishment methods on rice yields in a rice ratoon rice cropping system in Southwest China. Sci. Rep..

[bib35] Jiang S., Du B., Hu F., Zhang H., Kong P., Wu Q., Zhu J. (2022). A seven-year study on the effects of four tillage modes on soil physicochemical properties, microbial biomass, enzymatic activities, and grain yield in a ratoon rice cropping system.. Food Energy Secur..

[bib36] Johnson J.M., Ibrahim A., Dossou-Yovo E.R., Senthilkumar K., Tsujimoto Y., Asai H., Saito K. (2023). Inorganic fertilizer use and its association with rice yield gaps in sub-Saharan Africa. Glob. Food Secur..

[bib37] Katsura K., Maeda S., Lubis I., Horie T., Cao W., Shiraiwa T. (2008). The high yield of irrigated rice in Yunnan, China: ‘A cross-location analysis’. Field Crops Res..

[bib38] Kennedy P.L., Schmitz A., Linscombe S., Zhang F. (2022). The impact of cultivar development and cultural practices on Louisiana rice yield. Agrosystems, Geosci. Environ..

[bib39] Kiniry J.R., McCauley G., Xie Y., Arnold J.G. (2001). Rice Parameters describing crop performance of four U.S. Cultivars. Agron. J..

[bib40] Kouakou O., Mamadou C., Brahima K., Dick E., Konan K.F. (2014). Growth, yields and ratooning performance of lowland rice NERICA L14 as affected by different fertilizers. Indian J. Sci. Res. Technol..

[bib41] Kumar M., Das A., Layek J., Buragohain J., Idapuganti R.G., Babu S., Yadav G.S., Krishnappa R., Devi M.T. (2021). Impact of varieties and organic nutrient sources on productivity, soil carbon stocks and energetics of rice-ratoon system in Eastern Himalayas of India. Carbon Manag..

[bib42] Kumar V., Ladha J.K. (2011). Direct seeding of rice: recent developments and future research needs. Adv. Agron..

[bib43] Lal B., Gautam P., Nayak A.K., Raja R., Panda B.B., Tripathi R., Shahid M., Chatterjee D., Bhattacharyya P., Bihari P., Singh T., Meena S.K., Yadav V.K., Rathore V.S. (2023). Agronomic manipulation in main season and ratoon rice influences growth, productivity, and regeneration ability in tropical lowlands. Field Crops Res..

[bib44] Li S., Zhang Y., Guo L., Li X. (2022). Impact of tillage and straw treatment methods on rice growth and yields in a rice-ratoon rice cropping system. Sustainability.

[bib45] Ling X., Zhang T., Deng N., Yuan S., Yuan G., He W., Cui K., Nie L., Peng S., Li T., Huang J. (2019). Modelling rice growth and grain yield in rice ratooning production system. Field Crops Res..

[bib46] Liu X., Feng D., Yu G., Zhao H., Qiao L., Li Y., Fan X., Liu M., Zhang Q. (2016). Effect of different sowing dates in south Henan’s rice-growing areas on the growth and yield of ratoon rice.. Asian Agric. Res..

[bib47] Munda G.C., Das A., Patel D.P. (2009). Evaluation of transplanted and ratoon crop for double cropping of rice (*Oryza sativa* L.) under organic input management in mid altitude sub-tropical Meghalaya. Curr. Sci..

[bib48] Nakano H., Tanaka R., Wada H., Okami M., Nakagomi K., Hakata M. (2020). Breaking rice yield barrier with the ratooning method under changing climatic conditions: A paradigm shift in rice-cropping systems in southwestern Japan. Agron. J..

[bib49] Nakano H., Tanaka R., Nakagomi K., Hakata M. (2021). Grain yield response to stubble leaf blade clipping in rice ratooning in southwestern Japan. Agron. J..

[bib50] Nakano H., Tanaka R., Hakata M. (2022). Non-structural carbohydrate content in the stubble per unit area regulates grain yield of the second crop in rice ratooning. Crop Sci..

[bib51] Nakano H., Tanaka R., Hakata M. (2023). Grain yield response to planting date and cutting height of the first crop in rice ratooning. Crop Sci..

[bib52] Pasaribu P.O., Triadiati, Anas I. (2018). Rice Ratooning Using the Salibu System and the System of Rice Intensification Method Influenced by Physiological Traits. Pertanika J. Trop. Agric. Sci..

[bib53] Peng S., Zheng C., Yu X. (2023).

[bib54] Pittelkow C.M., Linquist B.A., Lundy M.E., Liang X., Van Groenigen K.J., Lee J., Van Gestel N., Six J., Venterea R.T., Van Kessel C. (2015). When does no-till yield more? A global meta-analysis. Field Crops Res..

[bib55] Ringera T.J., Kinyamario I.J., Amugune O.N., Kanya I.J. (2014). Ratooning ability and its effect on grain yield of upland Nerica rice varieties in Central Kenya. Int. J. Agron. Agric. Res..

[bib56] Sacks E.J., Dhanapala M.P., Tao D.Y., Cruz M.S., Sallan R. (2006). Breeding for perennial growth and fertility in an *Oryza sativa*/*O. longistaminata* population. Field Crops Res..

[bib57] Saito K., Senthilkumar K., Dossou-Yovo E.R., Ali I., Johnson J.M., Mujawamariya G., Rodenburg J. (2023). Status quo and challenges of rice production in sub-Saharan Africa. Plant Prod. Sci..

[bib58] Samson B.K., Voradeth S., Zhang S., Tao D., Xayavong S., Khammone T., Douangboupha K., Sihathep V., Sengxua P., Phimphachanhvongsod V., Bouahom B. (2018). Performance and survival of perennial rice derivatives (*Oryza sativa* L./*Oryza longistaminata*) in Lao PDR. Exp. Agric..

[bib59] Sanna V.A., Kassoh F.A., Bah A.M. (2018). Screening lowland rice genotypes for ratooning performance in the associated mangrove swamp of Sierra Leone. Int. J. Agric. Innov. Res..

[bib60] Sanni K.A., Ojo D.K., Adebisi M.A., Somado E.A., Ariyo O.J., Sie M., Akintayo I., Tia D.D., Ogunbayo S.A., Cisse B., Sikirou M., Adekoya M.A. (2009). Ratooning potential of interspecific NERICA rice varieties (*Oryza glaberrima* x *Oryza sativa*). Int. J. Bot..

[bib61] Satapathy B.S., Chatterjee D., Saha S., Duary B., Singh T. (2022). Weed management in a direct-seeded rice-ratoon rice cropping system. J. Agric. Sci..

[bib62] Setiawan A., Tyasmoro Y.S., Nugroho A. (2014). Intermittent irrigation and cutting height on growth and yield ratoon rice (*Oryza sativa* L.). *Agrivita*.

[bib63] Sha X.Y., Linscombe S.D., Groth D.E. (2007). Field evaluation of imidazolinone-tolerant Clearfield rice (*Oryza sativa* L.) at nine Louisiana locations. Crop Sci..

[bib64] Shamiul Islam M., Hasanuzzaman M., Rokonuzzaman Md (2008). Ratoon rice response to different fertilizer doses in irrigated condition. Agric. Conspec. Sci..

[bib65] Shiraki S., Cho T.M., Htay K.M., Yamaoka K. (2020). Effects of the double-cutting method for ratooning rice in the SALIBU system under different soil moisture conditions on grain yield and regeneration rate. Agronomy.

[bib66] Shiraki S., Cho T.M., Matsuno Y., Shinogi Y. (2021). Evapotranspiration and crop coefficient of ratoon rice crop determined by water depth observation and Bayesian inference. Agronomy.

[bib67] Sinaga P.H., Trikoesoemaningtyas, Didy S., Hajrial A. (2014). Screening of rice genotypes and evaluation of their ratooning ability in tidal swamp area. Asian J. Agric. Res..

[bib68] Song K., Zhang G., Yu H., Huang Q., Zhu X., Wang T., Xu H., Lv S., Ma J. (2020). Evaluation of methane and nitrous oxide emissions in a three-year case study on single rice and ratoon rice paddy ﬁelds. J. Clean. Prod..

[bib69] Song K., Zhang G., Yu H., Hua X., Lv S., Ma J. (2021). Methane and nitrous oxide emissions from a ratoon paddy field in Sichuan Province, China. Eur. J. Soil Sci..

[bib70] Song K., Zhang G., Ma J., Peng S., Lv S., Xu H. (2022). Greenhouse gas emissions from ratoon rice fields among different varieties. Field Crops Res..

[bib71] Tanaka R., Hakata M., Nakano H. (2021). Grain yield response to cultivar and harvest time of the first crop in rice ratooning in southwestern Japan. Crop Sci..

[bib72] Torres R.O., Natividad M.A., Quintana M.R., Henry A. (2020). Ratooning as a management strategy for lodged or drought-damaged rice crops. Crop Sci..

[bib73] Wang W., He A., Jiang G., Sun H., Jiang M., Man J., Ling X., Cui K., Huang J., Peng S., Nie L. (2020). Ratoon rice technology: A green and resource-efficient way for rice production. *Adv. Agron.* 159.

[bib74] Wang Y., Zheng C., Xiao S., Sun Y., Huang J., Peng S. (2019). Agronomic responses of ratoon rice to nitrogen management in central China. Field Crops Res..

[bib75] Wang Y., Li X., Tarpley L., Peng S., Dou F. (2021). Effects of nitrogen management on the ratoon crop yield and head rice yield in South USA. J. Integr. Agric..

[bib76] Weng P., Yang S., Pang Z., Huang J., Shen L., Letuma P., Chen H., Lin W. (2023). Effects of the configurations with different organic and inorganic fertilizers on grain yield and its related physiological traits of the main and its ratoon rice crops. Technol. Agron..

[bib77] Xia F., Wang W., Weng Y., Ali I., Zhao J., Nie Z., Li X., Yao X., Yang T. (2022). Productivity and water use of ratoon rice cropping systems with water-saving, drought-resistant rice. Agron. J..

[bib78] Xu Y., Liang L., Wang B., Xiang J., Gao M., Fu Z., Long P., Luo H., Huang C. (2022). Conversion from double-season rice to ratoon rice paddy ﬁelds reduces carbon footprint and enhances net ecosystem economic beneﬁt. Sci. Total Environ..

[bib79] Yamaoka K., Ofori J. (2020). Changing the nervous mind of small-scale farmers who are reluctant to invest for their development. Irrig. Drain..

[bib80] Yang D., Peng S., Zheng C., Xiang H., Huang J., Cui K., Wang F. (2021). Effects of nitrogen fertilization for bud initiation and tiller growth on yield and quality of rice ratoon crop in central China. Field Crops Res..

[bib81] Yang D., Peng S., Zheng C., Xiong Z., Yang G., Deng S., Wang F. (2022). Stubble height affects the grain yield of ratoon rice under rainfed conditions. Agric. Water Manag..

[bib82] Yao F., Hu Q., Yu Y., Yang L., Jiao S., Huang G., Zhang S., Hu F., Huang L. (2022). Regeneration pattern and genome-wide transcription profile of rhizome axillary buds after perennial rice harvest. Front. Plant Sci..

[bib83] Ying J., Peng S., He Q., Yang H., Yang C., Visperas R.M., Cassman K.G. (1998). Comparison of high-yield rice in tropical and subtropical environments: I. Determinants of grain and dry matter yields. Field Crops Res..

[bib84] Yu X., Guo Y., Yang G., Zhang Z., Liang Y., Zheng C., Xu L., Yuan S., Wang F., Huang J., Peng S. (2022). Nitrogen response of regenerated tillers varied among node positions in ratoon rice. Field Crops Res..

[bib85] Yuan S., Cassman K.G., Huang J., Peng S., Grassini P. (2019). Can ratoon cropping improve resource use efficiencies and profitability of rice in central China?. Field Crops Res..

[bib86] Yuan S., Linquist B.A., Wilson L.T., Cassman K.G., Stuart A.M., Pede V., Miro B., Saito K., Agustiani N., Aristya V.E., Krisnadi L.Y. (2021). Sustainable intensification for a larger global rice bowl. Nat. Commun..

[bib87] Zarwazi L.M., Junaedi A., Sopandie D., Sugiyanta Purwono, Agustiani N., Sujinah Nildalina (2021). Agronomic performance of rice varieties in modified ratoon salibu. IOP Conf. Ser.: Earth Environ. Sci..

[bib88] Zhang L., Jiang P., Guo X., Zhou X., Zhu Y., Liu M., Xiong H., Xu F. (2019). Integrated water and nitrogen management practices to enhance yield and environmental goals in rice-ratoon rice systems. Agron. J..

[bib89] Zhang L., Tang Q., Li L., Xu H., Zheng H., Wang J., Hua Y., Ren L., Tang J. (2022). Ratoon rice with direct seeding improves soil carbon sequestration in rice fields and increases grain quality. J. Environ. Manag..

[bib90] Zhang Q., Liu X., Yu G., Wang H., Feng D., Zhao H., Liu L. (2021). Agronomic and physiological characteristics of high-yielding ratoon rice varieties. Agron. J..

[bib91] Zhang Q., Liu X., Yu G., Duan B., Wang H., Zhao H., Feng D., Gu M., Liu L. (2022). Reasonable nitrogen regime in the main crop increased grain yields in both main and ratoon rice. Agriculture.

[bib92] Zhang Q., Liu X., Yu G., Duan B., Wang H., Zhao H., Feng D., Gu M., Hu Y., Chen Y., Liu L. (2022). Alternate wetting and moderate soil drying could increase grain yields in both main and ratoon rice crops. Crop Sci..

[bib93] Zhang S., Hu J., Yang C., Liu H., Yang F., Zhou J., Samson B.K., Boualaphanh C., Huang L., Huang G., Zhang J., Huang W., Tao D., Harnpichitvitaya D., Wade L.J., Hu F. (2017). Genotype by environment interactions for grain yield of perennial rice derivatives (*Oryza sativa* L./*Oryza longistaminata*) in southern China and Laos. Field Crops Res..

[bib94] Zhang S., Huang G., Zhang Y., Lv X., Wan K., Liang J., Feng Y., Dao J., Wu S., Zhang L., Yang X., Lain X., Huang L., Shao L., Zhang J., Qin S., Tao D., Crews T., Sacks E.J., Lyu J., Wade L.J., Hu F. (2023). Sustained productivity and agronomic potential of perennial rice. Nat. Sustain..

[bib95] Zhang Y., Tang Q., Zou Y., Li D., Qin J., Yang S., Chen L., Xia B., Peng S. (2009). Yield potential and radiation use efficiency of “super” hybrid rice grown under subtropical conditions. Field Crops Res..

[bib96] Zhang Y., Huang G., Zhang S., Zhang J., Gan S., Cheng M., Hu J., Huang L., Hu F. (2021). An innovated crop management scheme for perennial rice cropping system and its impacts on sustainable rice production. Eur. J. Agron..

[bib97] Zheng C., Wang Y., Yuan S., Yu X., Yang G., Yang C., Yang D., Wang F., Huang J., Peng S. (2022). Effects of skip-row planting on grain yield and quality of mechanized ratoon rice. Field Crops Res..

[bib98] Zheng C., Wang Y., Xu W., Yang D., Yang G., Yang C., Huang J., Peng S. (2023). Border effects of the main and ratoon crops in the rice ratooning system. J. Integr. Agric..

[bib99] Zheng H., Zhou W., Chen Q., Chen Y., Tang Q. (2019). Water-saving irrigation practices for rice yield information and nitrogen use efﬁciency under sub-tropical monsoon climate. Water Supply.

[bib100] Zhou Y., Liu K., Harrison M.T., Fahad S., Gong S., Zhu B., Liu Z. (2022). Shifting rice cropping systems mitigates ecological footprints and enhances grain yield in central China. Front. Plant Sci..

